
JOURNAL INFORMATION
==============================
NLM Title Abbreviation: Exp Physiol
Journal ID: Exp Physiol
NLM Journal ID: 9002940
Journal ID: EPH

Experimental Physiology

ISSN: 0958-0670
EISSN: 1469-445X
Publisher: Wiley

ARTICLE INFORMATION
==============================
PMCID: PMC13533522
DOI: 10.1113/eph.70450
Article ID: EPH70450
Article version: 1
Subjects: Issue Information, Issue Information

Issue Information

Electronic publication date: 2026 Sep 1
Publication date: 2026 Sep
Volume: 111
Issue: 9
Issue title: Special Issue: Space physiology: challenges and solutions for a journey to Mars
First page: 4250
Last page: 4254
Copyright: © 2026 The Authors. Experimental Physiology published by John Wiley & Sons Ltd on behalf of The Physiological Society.
License: This is an open access article under the terms of the http://creativecommons.org/licenses/by/4.0/ License, which permits use, distribution and reproduction in any medium, provided the original work is properly cited.
License URL: https://creativecommons.org/licenses/by/4.0/

Figure count: 0
Table count: 0
Page count: 5

source-schema-version-number: 2.0
cover-date: 1 September 2026
details-of-publishers-convertor: Converter:WILEY_ML3GV2_TO_JATSPMC version:6.8.6 mode:remove_FC converted:01.09.2026

==============================

### Experimental Physiology

Open Access

### Space physiology: challenges and solutions for a journey to Mars

Volume 111 | Issue 9 | 1 September 2026

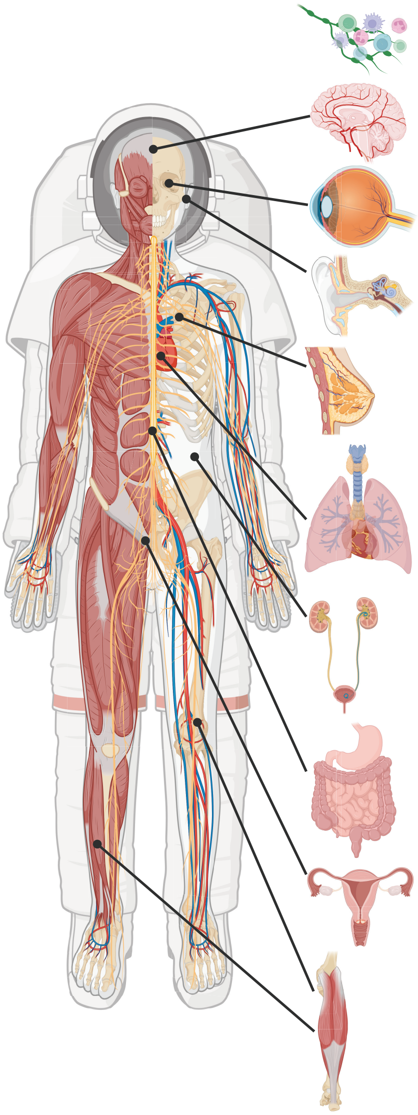

WILEY

The Physiological Society

150 YEARS 1876 2026 LEADING DISCOVERY SCIENCE

### Experimental Physiology

Open Access

Integration and Translation

Volume 111/Issue 9/
1 September 2026

### EDITORIAL BOARD

**EDITOR-IN-CHIEF**
Damian Bailey *Pontypridd, UK*

**DEPUTY EDITOR-IN-CHIEF EUROPE**
Ronan Berg *Copenhagen, Denmark*

**DEPUTY EDITOR-IN-CHIEF USA**
David Poole *Manhattan, KS, USA*

**SENIOR ETHICS EDITOR**
Susan Barman *East Lansing, MI, USA*

**STATISTICAL EDITOR**
Cody Durrer *Copenhagen, Denmark*

**SENIOR REVIEWS EDITOR**
Toby Mündel *Ontario, Canada*

**CONNECTIONS EDITOR**
Prem Kumar *Birmingham, UK*

**SENIOR EDITORS**
Dana Almohazey *Dammam, Saudi Arabia*
Jill Barnes *Madison, WI, USA*
R. Matthew Brothers *Arlington, TX, USA*
Kanokwan 'Mandy' Bunsawat *Iowa City, IA, USA*
Kirstene Callø *Copenhagen, Denmark*
Katrina Colafella *Victoria, Australia*
Ellen Dawson *Liverpool, UK*
Colleen Deane *Southampton, UK*
Peter Zu Eulenburg *Munich, Germany*
Federico Formenti *London, UK*
Shigehiko Ogoh *Kawagoe, Japan*
Jason Peart *Gold Coast, Australia*
Peter Rasmussen *Copenhagen, Denmark*
Lee Romer *London, UK*
Harry B. Rossiter *Los Angeles, CA, USA*
Bill Sheel *Vancouver, Canada*
Mike Stembridge *Cardiff, UK*

**REVIEWING EDITORS**
Tatsuro Amano *Niigata, Japan*
Claire Badenhorst *Palmerston North, NZ*
Tracy Baynard *Boston, MA, USA*
Hannah Caldwell *Copenhagen, Denmark*
Silvia Conde *Lisbon, Portugal*
Joseph Costello *Portsmouth, UK*
Jennifer Davies *Cardiff, UK*
Aimee Drane *Cardiff, UK*
Andrea Fellet *Buenos Aires, Argentina*
Carrie Ferguson *Torrance, CA, USA*
Igor Fernandes *Niteroi, Brazil*
Ryan Hoiland *Kelowna, Canada*
Seth Holwerda *Kansas City, KS, USA*
Anna Hudson *Adelaide, Australia*
Matthew Kay *Washington, DC, USA*
James Lang *Ames, Iowa, USA*
Jacqueline Limberg *Columbia, MO, USA*
Fiona McBryde *Auckland, NZ*
Carmen De Miguel *Birmingham, AL, USA*
Niels Olesen *Copenhagen, Denmark*
Leah Reznikov *Gainesville, FL, USA*
Benjamin Ryan *Boston, MA, USA*
Kleiton Silva *Camden, NJ, USA*
Sarah Short *Iowa City, IA, USA*
Can Ozan Tan *Boston, MA, USA*
Mike Tymko *Vancouver, Canada*
Song Yao *Melbourne, Australia*
Gensheng Zhang *Hangzhou, China*

**FUTURE LEADERS**
Edward Ashworth *Basel, Switzerland*
Sophie Broome *Melbourne, Australia*
Callum Brownstein *Newcastle, UK*
Daniel Craighead *Minneapolis, MN, USA*
Robert Lakin *Toronto, Canada*
Jedd Pratt *Manchester, UK*
Fabio Sarto *Padua, Italy*
Hollie Speer *Canberra, Australia*

**CONSULTANT EDITORS**
Richard Burden *Bisham, UK*
Nic Green *Bedfordshire, UK*
Julie Greeves *Salisbury, UK*
Denis Noble *Oxford, UK*
Nanduri Prabhakar *Chicago, IL, USA*

**PUBLISHER**
Alex Stewart (astewart@physoc.org)

**DEPUTY MANAGING EDITOR**
Hollie Evans (hevans@physoc.org)

**PEER REVIEW MANAGER**
Diana Jones (ephjournal@physoc.org)

**PEER REVIEW COORDINATOR**
Paroma Guha (ephjournal@physoc.org)

**EDITORIAL OFFICE**
The Physiological Society
Hodgkin Huxley House
30 Farringdon Lane, Clerkenwell
London EC1R 3AW

**PRODUCTION OFFICE**
Aakriti Saini (EPH@wiley.com)

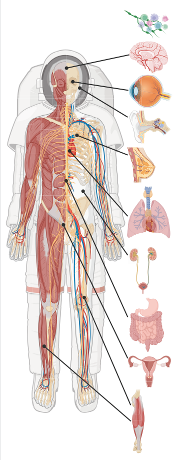

**Cover Illustration**
Known physiologic systems in women impacted by the spaceflight environment. More systems may be impacted; however, those systems are understudied. See Hughes-Fulford et al. Pages 3944–3967.

**Open Access and Copyright**
All articles published by Experimental Physiology [submitted after 18 October 2022] are fully open access: immediately freely available to read, download, and share. All articles [accepted from 18 October 2022] are published under the terms of a Creative Commons license.
Copyright on any research article published by Experimental Physiology is retained by the author(s).
Further information about open access licenses and copyright can be found at: https://authorservices.wiley.com/author-resources/Journal-Authors/licensing/licensing-info-faqs.html#4. Information on the licenses used by Experimental Physiology can be found at: https://physoc.onlinelibrary.wiley.com/journal/1469445X.

For submission instructions and all other information visit: https://physoc.onlinelibrary.wiley.com/journal/1469445X

The Physiological Society and The Physiological Society logo are trade marks belonging to The Physiological Society and are registered in the UK and in the EU Community, respectively.

*Experimental Physiology* publishes high quality, original, physiological research papers that give novel insights into fundamental homeostatic and adaptive responses in health and papers that further our knowledge of pathophysiological mechanisms in diseases.

Experimental Physiology
Open Access
Integration and Translation

### INFORMATION AND GUIDANCE FOR AUTHORS

**Full version online at http://ep.msubmit.net/cgi-bin/main.plex?form_type=display_requirements**

### ORIGIN

*The Quarterly Journal of Experimental Physiology*, founded in 1908 by Sir Edward Sharpey-Schafer, Professor of Physiology, University of Edinburgh, became a publication of The Physiological Society in 1981. The title was changed to *Experimental Physiology* in 1990.

### SCOPE

*Experimental Physiology* publishes research papers that report novel insights into homeostatic and adaptive responses in health, as well as those that further our understanding of pathophysiological mechanisms in disease. We encourage papers that embrace the journal's orientation of translation and integration, including studies of the adaptive responses to exercise, acute and chronic environmental stressors, growth and aging, and diseases where integrative homeostatic mechanisms play a key role in the response to and evolution of the disease process. Examples of such diseases include hypertension, heart failure, hypoxic lung disease, endocrine and neurological disorders. We are also keen to publish research that has a translational aspect or clinical application. Comparative physiology work that can be applied to aid the understanding human physiology is also encouraged.

Manuscripts that report the use of bioinformatic, genomic, molecular, proteomic and cellular techniques to provide novel insights into integrative physiological and pathophysiological mechanisms are welcomed.

We are also interested in methodological papers reporting important new developments in physiological techniques that could lead to significant new insights.

Papers which observe a significant new physiological behaviour or phenomenon which has the potential to alter the understanding of that field, but which have not yet identified the mechanisms may also be considered. Such papers should demonstrate the experimental examination and exclusion of a number of plausible mechanisms for the important novel phenomenon.

### Article Types

In addition to original research, *Experimental Physiology* consider the following article types for publication:

*Case studies, Short Communication, Connections, Reviews, Full Reviews, Hot Topic Reviews Myths and Methodologies and Symposium Reports.*

Review articles are normally by invitation but proposals may be submitted for consideration.

Commentary articles in the form of Viewpoints and Letters are also published.

For full guidelines on all published article see full guidelines.

C O P E COMMITTEE ON PUBLICATION ETHICS

This journal is a member of and subscribes to the principles of the Committee on Publication Ethics

### ETHICS OF EXPERIMENTATION

**Animal Experiments** - see full journal policy at https://physoc.onlinelibrary.wiley.com/hub/animal-experiments

Experimental Physiology endorses the ARRIVE Guidelines for reporting *in vivo* experiments: http://onlinelibrary.wiley.com/doi/10.1113/expphysiol.2010.053793/full

Authors of papers submitted to Experimental Physiology must adhere to the journals' principles and standards for reporting animal experiments.

Authors should read these guidelines to ensure that their research and experiments adhere to our exacting standards.

Euthanasia/humane killing: Animals must be killed using methods approved for that species, stage of development and size. The standards required by the journals are those of the UK/European Union legislation, set out in in ASPA Schedule 1 in the UK and in Annex IV in the European Directive 2010/63/EU. Methods not on Schedule 1 or Annex IV could be permissible but even where the method used conforms with national guidelines, the onus is on the author to provide scientific evidence to demonstrate that it is at least as humane as those on Schedule 1 and Annex IV.

Our guidelines have been outlined in the Editorial 'Principles and standards for reporting animal experiments in Experimental Physiology'.

**Confirmation of compliance**

Authors must state in the Methods section that the investigators understand the ethical principles under which the journal operates and that their work complies with the animal ethics checklist as outlined in the Editorial.

**Human Experiments** - See full journal policy at https://physoc.onlinelibrary.wiley.com/hub/human-experiments

The Editorial Boards are concerned that all work published in The Journal of Physiology and Experimental Physiology should have been conducted according to the highest standards of safety and ethics. Legislation and accepted practice concerning human experimentation vary from country to country and consequently it is difficult to define absolute requirements. However, work with human subjects should conform to the standards set by the Declaration of Helsinki (last modified in 2013), the Medical Research Council's online guidance on the use of human tissue, and the guidelines set out below. The objectives must be to ensure that all risks are minimized and that subjects are not injured and do not feel they have been abused as a result of participating in the study. Any definition of abuse will include excessive or unexpected pain or discomfort experienced during the experiment. Note, in the case of experiments involving minors, any risks must have been considered absent or minimal, and evidence must be presented that the experiments were performed with the understanding and consent of the legal guardian.

All manuscripts must contain statements indicating that (i) informed consent has been obtained, preferably in writing, (ii) studies conformed to the standards set by the Declaration of Helsinki or the version that was in place at the time of the experiments, NOTE: If the research study was registered (clause 35 of the Declaration of Helsinki) the registration database should be indicated, else the lack of registration should be noted as an exception (e.g. The study conformed to the standards set by the Declaration of Helsinki, except for registration in a database.); and (iii) the procedures have been approved by the local ethics committee.

### DISCLAIMER

The Publisher, the Physiological Society and Editors cannot be held responsible for any errors in or any consequences arising from the use of information contained in this journal. The views and opinions expressed do not necessarily reflect those of the Publisher, the Physiological Society or Editors, neither does the publication of advertisements constitute any endorsement by the Publisher, the Physiological Society, Editors, or Authors of the products advertised.

Wiley Open Access articles posted to repositories or websites are without warranty from Wiley of any kind, either express or implied, including, but not limited to, warranties of merchantability, fitness for a particular purpose, or non-infringement. To the fullest extent permitted by law Wiley disclaims all liability for any loss or damage arising out of, or in connection, with the use of or inability to use the content.

### Experimental Physiology

Vol 111.9

### Contents of volume 111.9

1 September 2026

### EDITORIAL

3863 Space physiology: Challenges and solutions for a journey to Mars
*Damian M. Bailey*

3870 Blistering barnacles: Space physiology in *The Adventures of Tintin*
*Jacob P. Hartmann, Mathis B. Mottelson, Rasmus H. Dahl, Ronni R. Plovsing and Ronan M. G. Berg*

3874 Beyond the horizon: Why space biology is the next great innovation opportunity
*Carina Kern and Keith Siew*

3877 Seizing opportunities and navigating the challenges of spaceflight research as an early career researcher
*Colleen S. Deane*

3880 Houston, we have a problem: Coagulation concerns during long-term spaceflight
*Lewis Fall and Damian M. Bailey*

3884 To Mars through LEO: How commercial space travel will change exploration-enabling research
*Christopher Puhl and Michail Magkos*

3890 The physiology of survival: Space
*Damian M. Bailey and Angelique van Ombergen*

3895 Space biology as a catalyst for the next biotech and longevity revolution
*Carina Kern, Matthew Cook, Elizabeth Reynolds, Anuj Ashar, Keith Siew, Ian R. D. Johnson, Nikodem Grzesiak, Louise Jopling, Meganne Christian, Rafal Libera, Immanuel Gartner, Miguel J. S. Ferreira, Aliasger K. Salem, William Birch, Nasreen Dhanji, David C. Cullen, Jorge M. Sampaio, Diogo Oliveira, Stuart Catchpole, Rosie Richardson, Dominykas Milašius and Damian Miles Bailey*

### VIEWPOINT

3902 Decoding the space integrome: Personalized countermeasures for a mission to Mars
*Damian M. Bailey*

3906 From Earth to orbit: How to preserve muscle health in space and bed rest
*Antonios Matsakas and Colleen Deane*

3908 Watching the eye with Mars in sight
*Peter zu Eulenburg, Lonnie Petersen and Damian M. Bailey*

3912 Entering a new era for exercise countermeasures in human spaceflight
*Enrico De Martino, Patrick Swain, Kirsty Lindsay, Claire Bruce-Martin, Ewoud Jacobs, Bradley Barbour, Chris Buckley, Adam C. McDonnell, Nick Caplan and Luke Hughes*

3916 Beyond intracranial pressure: Cerebrospinal fluid compartmentalization in spaceflight-associated neuro-ocular syndrome
*Susan P. Mollan and Damian M. Bailey*

### SHORT COMMUNICATION

3919 Low-dose X-ray radiation induces an adaptive response: A potential countermeasure to galactic cosmic radiation exposure
*Siena Edwards, Jessica Adams, Anastasia Tchernikov and John G. Edwards*

### CASE REPORT

3928 Blood flow restriction exercise during microgravity exposure in parabolic flight
*Yannick Laflamme, Etienne Chassé and Luke Hughes*

3936 NEX4EX – A novel exercise device enabling resistive, plyometric and sensorimotor training during deep-space missions: A case report
*Jonas Böcker, Jochen Zange, Markus Gruber, Guillaume Fau, Rainer Rawer, Sören Törholm, Arnaud Runge and Jörn Rittweger*

### REVIEW ARTICLE

3944 Women in space: A review of known physiological adaptations and health perspectives
*Millie Hughes-Fulford, Danielle J. Carroll, Heather C. M. Allaway, Bonnie J. Dunbar and Aenor J. Sawyer*

3968 Confined spaces in space: Cerebral implications of chronic elevations of inspired carbon dioxide and implications for long-duration space travel
*Jay M. J. R. Carr, Philip N. Ainslie and Trevor Day*

3983 The search for the ultimate exercise countermeasure to preserve crew health and ensure mission success in long-duration spaceflight
*Rodrigo Fernandez-Gonzalo, Stefan Schneider, Martina Heer and Anna-Maria Liphardt*

3993 Spaceflight and sport science: Physiological monitoring and countermeasures for the astronaut–athlete on Mars exploration missions
*Luke DeVirgiliis, Nicholas J. Goode, Kurt W. McDowell, Kirk L. English, Robert Novo, Virina Botros, Ginika Agwu, Jessica M. Scott and Lori L. Ploutz-Snyder*

4004 An eye on long-duration spaceflight: Controversies, countermeasures and challenges
*Vincent Wing Sum Ng and Susan Patricia Mollan*

4015 Spaceborne and spaceborn: Physiological aspects of pregnancy and birth during interplanetary flight
*Arun V. Holden*

4041 Isometric training at longer muscle–tendon complex lengths: A potential countermeasure to impaired neuro-muscle–tendon function during space travel
*Gerard McMahon, Andy Sanderson and Hans Degens*

4054 Translational perspectives on neurophysiological responses and mechanisms in blood flow restriction exercise
*Kai T. Fox, Luke Hughes, Brendan R. Scott, Paul S. R. Goods, Shaun YM. Teo and Ann-Maree Vallence*

4064 Tackling aging and chronic disease: How space exploration and the 'chameleon effect' of pharmaceuticals can lead the way
*Carina Kern, Tina Woods and Keith Siew*

### RESEARCH ARTICLE

### CARDIOVASCULAR CONTROL

4073 Jumping on the moon as a potential exercise countermeasure
*Patrick Swain, Filipa Santos, Luke Hughes, Dan Gordon and Nick Caplan*

4089 Haemodynamic responses to head-up tilt versus lower-body negative pressure in type 1 diabetes
*Victorien Faivre-Rampant, Jules Warnier, Perrine Larmet, Damien Garcia, Michael Joubert, Damian Miles Bailey, Igor Mekjavic, Hervé Normand and Amir Hodzic*

### ENVIRONMENTAL AND EXERCISE

4103 Blood flow restriction: The acute effects of body tilting and reduced gravity analogues on limb occlusion pressure
*Patrick Swain, Nick Caplan and Luke Hughes*

4115 Effects of the angle of head-down tilt on dynamic cerebral autoregulation during combined exposure to cephalad fluid shift and mild hypercapnia
*Tomokazu Kato, Yojiro Ogawa and Ken-ichi Iwasaki*

4126 50% body weight loading reduces stature increases and lumbar disc expansion from 4 h hyper-buoyancy floatation versus 15 min sitting upright
*David Marcos-Lorenzo, Christina Lysandrou, Laura Sudres, Alfonso Gil-Martinez, Jaap Swanenburg, James Edward Clark and David Andrew Green*

4140 Effect of artificial gravity on calcaneal bone marrow adipose tissue and mineral content in female and male participants in 60 days of bed rest
*Tammy Liu, Gerd Melkus, Tim Ramsay, Alain Berthiaume, Gabriele Armbrecht and Guy Trudel*

4153 ATHLETIC: An exoskeleton countermeasure exercise device for resistive and plyometric training in deep-space missions
*Jonas Böcker, Jochen Zange, Torsten Siedel, Guillaume Fau, Sebastian Langner, Thomas Krueger and Jörn Rittweger*

4166 Hyper-buoyancy flotation increases cervical disc height and reduces vertebral stiffness, with only partial reversal after acute 1 *g* axial loading
*D. Marcos-Lorenzo, P. Hernández Bernal, R. Fernández Corujo, A. Gil Martínez, J. Swanenburg and D. A. Green*

### MUSCLE

4179 Microgravity-induced changes in skeletal muscle and possible countermeasures: What we can learn from bed rest and human space studies
*Alessandra Bosutti, Bergita Ganse, Nicola A. Maffiuletti, Rob C. I. Wüst, Gustav J. Strijkers, Andy Sanderson and Hans Degens*

### NEUROSCIENCE

4195 An experimentally informed computational model of neurovestibular adaptation to altered gravity
*Victoria G. Kravets and Torin K. Clark*

4209 Physiological cerebrospinal fluid interactions between brain and eye structures are altered after long-duration spaceflight
*Ge Tang, Steven Jillings, Ben Jeurissen, Elena Tomilovskaya, Inna Nosikova, Alexandra Ryabova, Ekaterina Pechenkova, Viktor Petrovichev, Ilya Rukavishnikov, Stefan Sunaert, Paul M. Parizel, Lyudmila Makovskaya, Valentin Sinitsyn, Jan Sijbers, Jitka Annen, Steven Laureys, Angelique Van Ombergen, Seyed-Ahmad Ahmadi, Floris L. Wuyts and Peter zu Eulenburg*

### VASCULAR

4223 Regional changes in cerebral blood flow between the upright and supine posture and over 3 days of bed rest
*Carmen Possnig, Kyohei Marume, Gautam Babu, Sylvan L. J. E. Janssen, Christopher M. Hearon Jr, Katrin A. Dias, Satyam Sarma, Justin S. Lawley and Benjamin D. Levine*

4235 Cerebrovascular pulsatility following long duration spaceflight is associated with changes in pulse pressure and carotid artery stiffness
*Roxanne Fournier, Danielle K. Greaves, J. Kevin Shoemaker, Philippe Arbeille, Richard L. Hughson and Andrew D. Robertson*

### LETTER TO THE EDITOR

4248 The ultimate exercise countermeasure for long-duration spaceflight?
*Donald E. Watenpaugh and Alan R. Hargens*

Editor's Pick

Known physiologic systems in women impacted by the spaceflight environment. More systems may be impacted; however, those systems are understudied. See Hughes-Fulford et al. Pages 3944–3967.
